# A Retrospective Study on Pre- and Intraoperative Predictors on the Recovery Quality of Horses After General Anesthesia

**DOI:** 10.3390/vetsci12030262

**Published:** 2025-03-11

**Authors:** Bienvenida Román Durá, Oliver Dunham, Sigrid Grulke, Alexandra Salciccia, Julien Dupont, Charlotte Sandersen

**Affiliations:** 1 Department for Clinical Sciences of Equids, FARAH, ULiège, 4000 Liège, Belgium; b.roman@uliege.be (B.R.D.); sgrulke@uliege.be (S.G.); alexandra.salciccia@uliege.be (A.S.); julien.dupont@uliege.be (J.D.); 2 Department for Clinical Sciences of Companion Animals, FARAH, ULiège, 4000 Liège, Belgium; odunham@uliege.be

**Keywords:** anesthesia, equine, recovery, hypotension, hypoxemia, hypercapnia

## Abstract

Equine anesthesia is related to high morbidity and mortality rates and recent studies suggested that the period of recovery remains the phase associated with the greatest risk of mortality in horses. Intraoperative hypotension, hypoxemia, and hypercapnia are recognized as potential determinants of recovery quality. This study, conducted at the Equine University Hospital of Liege, aimed to explore how these factors influence recovery outcomes and compare complications between non-emergency and emergency procedures. We analyzed data from 1057 horses, with a mean weight of 498 kg (ranging from 150 to 850 kg) and a mean age of 10.7 (ranging from 0.5 to 37 years), undergoing standardized general anesthesia for non-emergency and emergency procedures to assess which factors influence recovery quality. Recoveries were graded as ‘good’ or ‘bad’ and age, sex, breed, American Society of Anesthesiologists (ASA) physical status classification, weight, anesthesia duration, presence of hypotension, hypoxemia, and hypercapnia were compared with a logistic regression analysis. Our findings highlight the multifactorial nature of anesthetic recovery in horses, with breed, age, anesthesia and recovery duration, and emergency status emerging as significant influencing factors. Additionally, monitoring and managing hypoxemia and hypotension remain critical due to their recognized impact on recovery quality. However, despite their clinical relevance, the specific effects of these variables on anesthetic recovery in horses remain insufficiently studied, emphasizing the need for further research to refine perioperative management strategies and improve outcomes.

## Simple Summary

Equine anesthesia is associated with a higher incidence of morbidity and mortality compared to other species, with the recovery phase representing the period of greatest risk. This study, conducted at the Equine University Hospital of Liege, retrospectively analyzed 1057 horses’ general anesthetic reports to identify factors influencing recovery quality, comparing non-emergency and emergency procedures. Using logistic regression, the impact of age, sex, breed, American Society of Anesthesiologists (ASA) physical status, body weight, anesthesia duration, and the presence of intraoperative hypotension, hypoxemia and hypercapnia on the recovery classification was evaluated. Variables that were found to be statistically significant in our study included age, breed, emergency procedures and the durations of anesthesia and recovery. These factors showed a notable correlation with the outcomes observed, suggesting that they play a crucial role in the recovery from general anesthesia in horses.

## 1. Introduction

Equine anesthesia is more likely to result in morbidity and mortality compared with other commonly anesthetized animal species [1]. In particular, the period of recovery following general anesthesia in horses remains the phase associated with the greatest risk of mortality [2]. A previous study reported an overall mortality rate of 1.9%, with mortality rates of 0.9% in non-colic cases and a markedly higher 7.8% in colic cases [3]. More recently, preliminary findings from the ongoing CEPEF-4 study have shown some improvement in these figures, although the overall mortality rate still stands at 1.0%. The study reported a mortality rate of 0.6% in horses undergoing non-colic procedures, while horses undergoing an exploratory laparotomy for colic had a significantly higher mortality rate of 3.4% [4]. Compared to horses, the mortality rates in dogs and cats are 0.05% and 0.11%, respectively, according to Matthews et al. (2017) [5].

Horses respond to anesthesia in a qualitatively similar manner to other species. However, their temperament, size, weight, expression of pain, and anatomy increase the likelihood and severity of several anesthesia-related pathophysiological, neuromuscular, and orthopedic issues, which are less common in other domestic animals [6]. As horses regain consciousness, they often attempt to stand and can exhibit signs of agitation. Due to their fight-or-flight instinct when sensing danger, horses are inclined to prematurely attempt rising from a lying position, consequently impacting the quality of recovery due to enduring ataxia and weakness [7].

The CEPEF-2 study revealed that 33% of deaths during surgery were due to intraoperative cardiac arrest or cardiovascular collapse, while fractures and myopathy during recovery accounted for another 32% of fatalities [3]. More recent reports indicate that the majority of deaths (81–100%) now occur during the recovery phase [8]. This shift in the timing of fatalities might be attributed to several factors, including changes in the drugs used for inducing anesthesia and the switch from halothane to sevoflurane or isoflurane [6]. Additionally, there have been significant improvements in monitoring equipment and techniques, as well as advancements in the training of anesthetists [8].

The risk factors that contribute to an unsafe recovery include a higher body mass, breeds such as Draft horses with increased height and muscle mass, temperament, advancing age, prolonged anesthesia duration, out-of-hours surgery, the presence of pain, the type of surgical procedure, the position during recumbency, medications administered intraoperatively, acid–base imbalances, physiological parameters, emergency situations, the availability of recovery assistance, health status evaluated by the American Society of Anesthesiologists (ASA) scale, and human error [6,7,9,10,11].

As suspected, in addition to equine-patient-determined factors, intraoperative factors, such as hypotension, hypoxemia, hypercapnia, hypothermia, or prolonged anesthesia duration, and the administration of certain drugs prior to the recovery phase can significantly impact the quality of recovery [2,12,13,14]. However, there have been few clinical studies documenting these correlations and they have not always reported the same results [1,15].

Hypotension, defined as a mean arterial pressure (MAP) of <70 mmHg, commonly occurs during general anesthesia using halogenated anesthetic agents in horses, with reported frequencies of 42% during elective procedures and up to 88% during abdominal surgeries [16,17,18]. Hypotension during surgery has been associated with extended periods prior to horses being able to stand up after anesthesia, diminished recovery quality, reduced survival rates in horses with acute abdominal disturbance, and a higher occurrence or severity of myopathy [19].

Hypoxemia during anesthesia is defined by an arterial partial pressure of oxygen (PaO_2_) below 80 mmHg [20]. When PaO_2_ values drop below 60 mmHg, hypoxemia is considered potentially life threatening. Reduced arterial oxygen content due to hypoxemia can lead to inadequate tissue oxygenation and improper low oxygen to maintain aerobic metabolism in the cells, resulting in hypoxia [21]. The incidence of hypoxemia in colic cases has been reported to be 13.6% [22]. Recently, a study revealed similar values for both elective and emergency cases, with an overall incidence of hypoxemia of 9.9% [21,23].

Hypercapnia is a common occurrence in anesthetized horses due to the respiratory depression induced by anesthetic agents. It is defined as a PaCO_2_ level exceeding 45 mmHg, and under certain conditions, it can be considered as a level of up to 70 mmHg [24]. Hypercapnia has not yet been sufficiently studied to determine its impact on the recovery of horses.

Several other factors may also influence anesthetic recovery in horses, but their effects have not yet been thoroughly studied. Further research is needed to better understand their role and clinical implications. The objective of this retrospective single-center study was to investigate the impact of pre- and intraoperative parameters on the quality of recovery from general anesthesia in horses.

## 2. Materials and Methods

### 2.1. Case Selection

This study constituted a single-center retrospective analysis conducted at the Equine University Hospital of Liege. The study included 1057 horses and ponies from different breeds undergoing general anesthesia between December 2020 and July 2024. Horses and ponies weighing over 150 kg and being at least 6 months old, for which complete information was available on the recovery quality and duration, were included. The owners provided consent for the use of medical files for research purposes upon their horse’s admission to the hospital. Information regarding the equine patient and specifics concerning anesthetic management, the recovery phase, and any complication after the procedure were acquired from the anesthesia report.

### 2.2. Case Management

Non-emergency cases were admitted to the hospital for a minimum of 12 h prior to surgery. They were allowed to drink water and eat hay but were fasted from concentrates for 12 h. Emergency cases were not fasted. Preanesthetic evaluations included clinical, hematological, and biochemical exams to determine the ASA status.

For all horses, a jugular vein was aseptically catheterized with a catheter (12 or 14 G) for the administration of drugs and fluids. Preoperative antibiotics and anti-inflammatory drugs (using non-steroidal anti-inflammatory drugs) were administered according to the surgeon’s preference. Horses for scheduled surgeries received 10 to 50 µg/kg of acepromazine (Tranquinervin, Le Vet Beheer, Oudewater, The Netherlands) IV or IM as a premedication where there was no specific contraindication. After achieving a proper level of sedation with an alpha-2-adrenoceptor agonist (xylazine (Nerfasin, Dechra, Bladel, The Netherlands) or detomidine (Domidine, Nerfasin, Dechra, Bladel, The Netherlands)), the induction was performed in all cases using ketamine (Ketamidor, VetViva Richter GmbH, Wels, Austria) 2.2–2.5 mg/kg IV and midazolam (Dormazolam, Dechra, Bladel, The Netherlands)) 0.04–0.06 mg/kg IV. The selection of premedication, induction agents, inhalant anesthetic (isoflurane or sevoflurane), and additional analgesics was guided by the anesthesiologist’s preference, considering the equine-patient’s history and concurrent conditions. In horses receiving total intravenous anesthesia (TIVA), anesthesia was maintained by an infusion of guaiphenesin with ketamine and xylazine for procedures expected to last less than 45 min and when aseptic conditions of the operating theatre were not required.

Horses’ tracheas were intubated. Subsequently, equine patients were placed on the table and positioned according to the planned procedure (lateral, dorsal, or sternal). The orotracheal or nasotracheal tube was connected to a large animal anesthetic circle system: Tafonius (Hallowell Engineering and Manufacturing Corp., Pittsfield, MA, USA) or Avante Titan XL (Avante Animal Health, Louisveille, KY, USA). Anesthesia was maintained using either isoflurane (IsoFlo^,^ Zoetis, Chatillon, France or sevoflurane (SevoTek Laboratoires Karizoo, Barcelona, Spain) or Sevoflo, Zoetis, Chatillon, France) delivered in oxygen or an oxygen/air mixture. Mechanical ventilation was promptly initiated with a tidal volume (TV, L) set at 10 mL/kg; a respiratory rate (RR, inspirations/minute) at 8–10 inspirations/minute, which was then adjusted to maintain an end-tidal carbon dioxide (ETCO_2_, mmHg) value between 35 and 55 mmHg; an inspiratory/expiratory ratio (I:E) of 1:2; and an inspired fraction of oxygen (FiO_2_) maintained at around 70%. A continuous rate infusion (CRI) of lactated Ringer’s solution was administered to all horses, starting at a rate of 5–10 mL/kg/hour. Fluid administration was continually adjusted with rates from 5–20 mL/kg/h based on the horse’s cardiovascular status, blood loss estimated from the volume recovered in the surgical suction reservoir and counting the swabs, and urinary output (through the placement of a urinary catheter, with urine recovered in a scaled bucket). Isoflurane and sevoflurane were titrated to maintain a sluggish palpebral reflex and, in some cases, xylazine was infused at rates of 0.3–0.6 mg/kg/hour. Based on the occurrence of nystagmus or sudden movements, horses were administered a bolus of ketamine (0.2–0.5 mg/kg IV) to deepen the plane of anesthesia. During surgery, additional analgesic support was occasionally supplemented with morphine or CRIs such as morphine, ketamine, or lidocaine.

A 20 G catheter was aseptically positioned in either the transverse facial, facial, auricular, or metatarsal artery, and connected to a pressure transducer through a fluid-filled line. The pressure transducer (Edwards Lifesciences, Gyancourt, France) was positioned at the level of the heart and zeroed to atmospheric pressure. The same catheter was also used to collect arterial blood samples. In instances where mean arterial pressure fell below 70 mmHg, a CRI of dobutamine (Dobutrex, Saint-Priest, France) or noradrenaline (Noradrenaline, Laboratoire Aguettant, Lyon, France) was administered to maintain a MAP within the 70 to 90 mmHg range. If the PaO_2_ fell below 100 mmHg, the TV was increased to 13 mL/kg, the RR was decreased to 7 breaths per minute, and an inspiratory pause of 30% of the inspiratory time was implemented. If no significant improvement was observed following these adjustments, inhaled salbutamol was administered at 2 µg/kg IT (Ventolin, GlaxoSmithKline Pharmaceuticals, Wavre, Belgium) and positive end-expiratory pressure was applied after an alveolar recruitment maneuver was performed.

### 2.3. Monitoring

Intraoperative parameters were monitored either with the integrated monitoring of the Tafonius anesthesia machine or with a stand-alone monitor (Datex S5, Helsinki, Finland) when anesthesia was delivered with the Titan anesthesia machine. The following parameters were recorded every five minutes: electrocardiography-derived heart rate, invasive arterial blood pressure, side-stream capnography, RR, TV, respiratory gas analysis (ETCO_2_ and FE_Iso_), measurement of FIO_2_, pulse oximetry (SpO_2_), and intraesophageal temperature. Additionally, assessments were made of nystagmus and the palpebral reflex, pulse quality, mucous membrane color, and capillary refill time. Blood gases, including measurements of pH, PaCO_2_, PaO_2_, SaO_2_, electrolytes (bicarbonate, Na^+^, K^+^, Cl^−^, and iCa^2+^), base excess (BE), and hematocrit, were analyzed in intervals of no more than 30 min. If deemed necessary by the anesthetist, lactate and glucose levels were also measured.

### 2.4. Recovery

Recovery was performed in a rubber-padded recovery box. During the recovery phase, horses requiring sedation were sedated with 0.2 mg/kg IV of xylazine. The dose of sedation could be increased at the discretion of the anesthetist. Throughout the recovery period, oxygen was supplied at a rate of 15 L per minute through the endotracheal tube. Extubation was performed once the horses were standing. Horses were assisted with ropes; one rope was attached to the halter and a second rope was tied to the tail. A separate person operated each rope. In certain cases, the Anderson sling was utilized, including those involving wounds with a cast or splint, as well as other clinical indications.

### 2.5. Intraoperative Complications

Each anesthetic record was thoroughly reviewed to identify intraoperative complications defined as follows: hypoxemia, consistent with the CEPEF criteria, as a PaO_2_ < 60 mmHg in at least one arterial blood gas analysis; hypotension, with a MAP < 70 mmHg persisting for at least 15 min; hypercapnia, as a PaCO_2_ > 60 mmHg in at least one arterial blood gas analysis; and hypothermia, as a body temperature of <35.5 °C at the conclusion of surgery.

### 2.6. Data Collection

As participants in the CEPEF-4 study, our hospital logged all case-related information into the CEPEF database. Data were retrospectively extracted from both the automatic electronic health record (Vetronic Services Limited, Devon, UK) and from the anesthesia records. Breeds were categorized into different groups: (1) Warmbloods (WB), (2) Racehorses (Race), such as Thoroughbred, Standardbred, and Arabians, (3) Draft-bred horses (Draft, Irish Cob, Tinker), (4) Ponies, (5) American horse breeds (USA, such as Quarter Horse, Paint, and Apaloosa) and Baroque Horses (such as Purebred Spanish Horse, Lusitano, Lipizzaner, and Frisian). Age (months), sex (stallion, gelding, pregnant mare, and non-pregnant mare), body mass (kg), and American Society of Anesthesiologists (ASA) physical status classification (1 to 5) were also recorded. The types of procedures included diagnostic procedures, colic surgery, non-colic abdominal surgery, fracture repair, orthopedic surgery, ophthalmologic surgery, urogenital surgery, surgery of ear/nose/throat (ENT), and miscellaneous surgery (e.g., dental procedures, wound debridement/treatment, eye enucleation, skin surgery, cast change, sarcoid removal, mandibular fracture, etc.).

The types of anesthesia used included total TIVA, partial intravenous anesthesia (PIVA), or inhalation-only anesthesia, along with the specific protocols employed. Additional recorded details comprised whether anesthesia was planned or conducted as an emergency procedure, the duration of anesthesia and surgery, recovery score (Table 1), recovery duration, documented complications, and the time of day during which the procedures took place. Body conditions scores were graded as thin, normal, semi-obese, and obese. Anesthesia time was defined as the interval from induction to discontinuation of the anesthetic agent, surgical time as the interval from the first incision to the final suture, and recovery time as the interval from discontinuation of the anesthetic to the final standing position.

### 2.7. Statistical Analysis

Once extracted, data were entered into a binary (yes/no) format in Microsoft Excel and categorized according to different cut-off values for recovery and imported into R (R Core Team 2024, R Foundation for Statistical Computing, Vienna, Austria) for analysis using the STATS package. Summary statistics were calculated in Microsoft Excel, and R and skewness were tested for significance with the D’Agostino test in the moments package. A logistic regression was then performed using a mixed linear model with a binomial method that included the collected variables, and the recovery was classified as either good and bad, based on a cut-off of 3, and more for the CEPEF-based recovery scores (Table 1). Variables with *p*-values greater than 0.1 were excluded for model refinement, and the analysis was repeated. The model deemed to have the most practical utility for dissemination was then selected and presented. Alpha and beta errors were set at 0.05 and 0.2, respectively, for the purposes of the analysis, with a *p*-value of <0.05 being considered significant. Results were presented in graphs and tables created using Microsoft Word and Excel.

## 3. Results

### 3.1. Demographic Data

A total of 1057 horses were included in the analysis, of which 873 (82.6%) experienced a good recovery, whereas 184 (17.4%) had a bad recovery (recovery score of 3 or greater). Four horses died during their recovery due to catastrophic limb fractures and one due to a cardiac arrest, and these were given a recovery score of 5. In the subsequent 6 days post-surgery, 31 more horses were euthanized, with the majority due to non-responsive colic at the owner’s request. Other problems leading to euthanasia included uncontrollable pain, severe lameness, the recurrence of colic, gastric rupture, neurological symptoms, a poor prognosis, diarrhea, an inability to stand, osteopenia, osteomyelitis, and cardiac arrest during hospitalization.

The demographic data, types of interventions, and intraoperative complications are summarized in Table 2. There were 211 stallions, 439 geldings, 392 non-pregnant mares, and 15 pregnant mares. Their age ranged from 6 months to 37 years with a mean age of 10.7 years and median age of 10.1 years. The breeds included 497 Warmbloods, 93 Baroque horses, 105 Racehorses, 89 Draft horses, 79 Ponies, 49 American horses, and 145 horses from other breeds. The mean weight was 498 kg (ranging from 150 kg to 850 kg).

### 3.2. ASA Physical Status Classification

The distribution of the horses based on the ASA scores was as follows: 162 horses were classified as having an ASA score of 1. The group with an ASA score of 2 included 628 horses. In the group with an ASA score of 3, there were 169 horses. The group with an ASA score of 4 encompassed 85 horses, and finally, those with an ASA score of 5 comprised 13 horses. As total of 771 horses (72.7%) underwent anesthesia for non-emergency surgery, while 289 horses (27.3%) were anesthetized in emergencies. No ASA or emergency status was significantly associated with a bad recovery.

### 3.3. Type of Procedure

This study involved a wide variety of procedures. Colic surgery was performed in 227 cases, while abdominal non-colic surgery was performed in 28 instances. Diagnostic, orthopedic, and ear–nose–throat-related procedures accounted for 9, 205, and 27 cases, respectively. Fracture repair was conducted in 12 cases. Urogenital procedures were performed in 171 cases. Ophthalmologic surgery and diagnostic procedures were performed in 185 horses. The remaining horses underwent surgical procedures that could not be attributed to any of the categories. In some cases, two or more procedures were performed at the same time.

### 3.4. Intraoperative Complications

Of the total of 1057 horses, 136 experienced hypoxemia as defined by the CEPEF criteria. Sixty-eight of these cases were emergencies. Salbutamol to treat hypoxemia was administered in 189 horses. Hypotension was diagnosed according to CEPEF criteria in 613 (60%) horses. Among those, 204 were attributed to emergencies such as colic cases. When hypotension occurred, horses received an infusion of dobutamine (663 cases). In four cases, the hypotension proved refractory to dobutamine, and an infusion of noradrenaline was administered as an alternative therapeutic approach. Hypercapnia was observed in 37 (3.50%) horses, with 17 cases occurring during emergency procedures including colic surgeries and 20 cases during non-emergency surgical procedures. In our review of all cases, we found that hypothermia (esophageal temperature sensor measurement of <35.5 °C degrees) was diagnosed in 97 horses of the 117 horses for which temperature was measured. Hypothermia was reported in 46 emergency cases, of which 39 cases were colic surgeries and 51 cases were non-emergency surgeries. Due to the low reporting, hypothermia was not included in the statistical analysis.

### 3.5. Duration of Anesthesia, Surgery, and Recovery

The procedures were characterized by a median anesthesia duration of 110 min, ranging from 15 to 340 min. The median surgical duration was 70 min, ranging from 5 to 600 min. The recovery duration was variable, with a median of 40 min ranging from 5 to 360 min. Recovery scores ranged from 1 to 5, with a mean score of 1.96, a median of 1, and a skew of 1.58 (*p* < 0.001) indicating that lower recovery scores are more likely. In the logistic regression, a cut-off recovery score of 3 or greater to indicate poor recovery was decided upon based on the outcomes of the different models.

### 3.6. Recovery Predictors

Factors associated with a poor recovery were the horse being an American breed when compared to Baroque horses; OR 2.74 (95% CI 1.04–7.33 *p* = 0.0436). No breeds were significantly different compared to the general population. Breed pairwise differences are summarized in Table 3. Additionally, the BCS was associated in a linear manner with recovery; OR 0.59 (95%CI 0.363–0.981 *p* = 0.0398), meaning horses with a lower body condition had a worse recovery.

#### Continuous Variables

Of the continuous variables, age (OR 1.04 per 10 years life (95% CI 1.01–1.07 *p* = 0.00276)), anesthesia duration (OR 1.41 per 60 min (95% CI 1.17–1.82, *p* = 0.00394)), and recovery time (OR 2.25 per 60 min recovery (95% CI 1.69–3.04 *p* = 0.0000007)) had increased recovery scores. Under this model, 11.6 h of anesthesia would be predicted to be the tipping point for a score of 3 or greater, rather than of 1 or 2.

## 4. Discussion

The successful recovery of horses from general anesthesia relies on several factors. Some of these factors can be managed by the anesthetist, while others are more challenging or even impossible to control [25]. The findings of this study suggest that a combination of preoperative and intraoperative variables may affect the quality of recovery, but that their exact impact is difficult to determine.

One of the major factors identified in this study was the duration of anesthesia, applicable to both emergency and non-emergency procedures. Consistent with our findings, existing research has shown that a prolonged duration of anesthesia has a detrimental impact on the quality of recovery in horses undergoing both non-emergency and emergency surgical procedures [13]. Prolonged anesthesia also increases the risk of post-operative complications and delays in recovery time. This effect is particularly significant in equine patients due to their unique physiology and susceptibility to anesthesia-related complications [26]. Recently, Brumund et al. have developed an accurate risk score called CHARIOT (Combined Horse Anesthetic Risk Identification and Optimization Tool), which predicts peri-anesthetic morbidity and mortality in equine patients. This advancement could significantly enhance perioperative management and improve outcomes [27].

In our study, a low body condition score (BCS) of 2–3/9 was identified as a borderline significant variable related to a bad recovery. This is in contrast with other studies, showing that a higher BCS is rather related to worse recovery. However, none of the horses in our study were identified as obese, which makes comparison difficult. Loomes (2024) identified horses with a BCS of 6/9 or higher as being at a greater risk of developing hypoxemia during dorsal recumbency for elective surgery [28]. Our study showed that body weight was not an indicator of recovery quality, although it has been shown before that horses weighing more than 550 kg appear to be nearly five times more likely to experience hypoxemia compared to those weighing less than 450 kg [23]. Anesthetized horses with rounder bellies have been shown to exhibit lower mean PaO_2_ values than those with flatter bellies, suggesting that body mass and conformation can influence oxygenation despite mechanical ventilation [21]. These findings underscore the significance of both low and high BCSs as potential contributors to anesthetic risk, recovery quality, and perioperative oxygenation.

Our study showed that older horses were statistically more likely to experience poorer anesthetic recoveries, but although statistically significant, this does not appear to be clinically relevant as the odds ratio increases by 1.14 for every ten years of age. Older horses are more susceptible to various health issues, including osteoarthritis, osteopenia, muscle atrophy, sarcopenia, and generalized weakness, which can negatively impact their ability to recover from anesthesia. Additionally, conditions such as pituitary pars intermedia dysfunction, commonly observed in geriatric horses, may further contribute to impaired recovery by affecting muscle integrity, metabolism, and overall resilience to physiological stress [29,30].

Another significant variable identified in this study was breed. Comparisons revealed that American breeds of horses exhibited poorer anesthetic recovery outcomes compared to Baroque horses. This difference may be attributed to variations in temperament, as behavioral factors are known to influence recovery quality. Horses exhibiting anxious or excitable temperaments showed worse quality recovery scores when contrasted with those displaying calm demeanors [10].

Surprisingly, other factors did not appear to significantly influence recovery quality. We would have expected that certain procedures, namely colic, wound/trauma, or fracture procedures would be related to a higher risk of a bad recovery. This is probably due to a multifactorial issue, involving pain, hemodynamic instability, and other contributing variables, all of which may influence anesthetic recovery and overall clinical outcomes in horses. Pain has been identified as a potential risk factor for a poorer-quality recovery in horses [10,31]. Horses demonstrating pain behavior during the preoperative period had an unsatisfactory recovery quality; these horses are probably less calm and may attempt to rise sooner, leading to recoveries with higher ataxia incoordination and muscular weakness [32,33]. Emergency abdominal surgeries and internal fracture fixation have been linked to higher mortality rates in various studies. This is likely due to the pain and extended anesthesia time needed for these more invasive procedures [9,15]. The increased mortality seen in horses with colic is thought to result from their pre-existing cardiovascular issues and higher ASA status, which indicate a more compromised health status before surgery [8].

Horses with colic frequently experience hypovolemia and endotoxemia due to intestinal damage, which can result in significant circulatory disturbances [34]. Hypotension commonly occurs during general anesthesia using volatile gases in horses, with reported frequencies of 42% during elective procedures and up to 88% during abdominal surgeries [16,17]. In adult horses, intraoperative hypotension has been closely associated with postoperative myopathy. Maintaining a mean arterial pressure above 70 mmHg has been demonstrated to reduce the incidence of myopathy. Various factors contribute to hypotension including myocardial depression, hypovolemia, changes in vascular resistance, and bradycardia [35,36]. In one study, horses requiring higher doses of dobutamine were found to be correlated with poor recoveries [19]. In our study, hypotension was not a significant variable, in contrast to most previous studies.

The same was observed for hypoxemia, where no link could be identified to the quality of recovery. Severe hypoxemia is often seen in horses undergoing surgery for colon torsion. In general, large intestinal lesions have shown three times the likelihood of hypoxemia compared to small intestinal lesions with reported improvements in PaO_2_ values following exteriorization of the colon [21,23]. Rüegg et al. (2016) demonstrated a higher risk of a poor recovery in horses with colic if hypoxemia occurred during anesthesia [14]. Additionally, Meier et al. (2023) showed that both hypoxemia and prolonged anesthesia duration negatively impact recovery quality [13]. Similar results were obtained in our study with hypoxemic horses that received salbutamol. In horses, hypoxemia has been associated with delayed cognitive recovery, potentially leading to prolonged recumbency in the recovery stall and consequent muscle weakness [20,37]. In our study, hypoxemia as defined by the CEPEF guidelines did not occur in many horses, as often horses were treated with adapted ventilation or salbutamol before the manifestation of severe hypoxemia. Salbutamol is a b_2_-adrenergic agonist that is an effective bronchodilator in horses with recurrent airway disease. The tracheal administration of a salbutamol aerosol significantly improves PaO_2_ in anaesthetized hypoxemic horses [38,39]. However, in our study, the administration of salbutamol did not demonstrate a statistically significant negative effect on recovery. In addition to inhaled salbutamol, various techniques are available to improve oxygenation, including controlled ventilation, an inspiratory pause of 30% of the inspiratory time, the application of positive end-expiratory pressure, and alveolar recruitment maneuver up to 80 cmH_2_O. Each of these methods plays a critical role in managing hypoxemia and optimizing respiratory function in clinical settings [40,41,42,43].

Blood gas measurement provides vital information about ventilation, oxygenation, and metabolic status [44,45]. In our study, the impact of hypercapnia on anesthetic recovery quality was not statistically significant. Further investigation is warranted to clarify the relationship between hypercapnia and recovery quality in equine anesthesia. It is possible that horses might tolerate moderate hypercapnia better than expected due to compensatory mechanisms such as increased oxygen delivery or improved muscle perfusion [44].

In non-emergency surgery, acepromazine is routinely administered due to its potential protective effect on recovery. The effect of acepromazine might still play a role during recovery after anesthesia of short-to-intermediate duration [2]. The results from the CEPEF-2 study suggest that the administration of acepromazine may play an important role in decreasing the risk of anesthesia-related mortality [3]. However, the impact of acepromazine administration during the perianesthetic period on recovery quality, morbidity, and mortality remains difficult to predict [10]. Furthermore, data from CEPEF-2 are not easily comparable to modern anesthesia practice, as halothane has largely been [26] replaced by isoflurane and sevoflurane. The ASA status score in our study did not appear to be statistically significantly related to recovery quality, which is in contrast to a previous study that showed a higher ASA physical status score has been associated with a diminished quality of recovery [46].

This retrospective study has several limitations. It did not include morbidities such as neuropathies, myopathies, and others in the analysis due to the inherent limitations of retrospective data collection and documentation. Additionally, variability in management protocols among different anesthetists posed another significant limitation affecting procedures ranging from premedication, maintenance, and recovery to the management of anesthesia and recovery complications. A major limitation was the absence of standardized protocols for managing perioperative complications. While some cases were treated according to CEPEF instructions, others followed institution-specific protocols, leading to discrepancies in case logging. Furthermore, the lack of standardized criteria for assessing recovery quality introduced subjectivity into the evaluation process.

One notable example of this variability is the discrepancy between the number of hypoxemic horses recorded and the number of treatments administered, likely due to differing definitions of hypoxemia. In our clinic, mild hypoxemia was defined as a PaO_2_ below 95 mmHg, whereas the CEPEF study defined hypoxemia as a PaO_2_ below 60 mmHg. This difference in classification may also reflect potential errors in data encoding. Additionally, several cases were excluded from the study due to incomplete or insufficiently documented anesthesia records, further limiting the dataset and its generalizability.

## 5. Conclusions

Our study revealed that increasing age, certain breeds, emergency procedures and the increasing length of anesthesia are statistically related to worse recoveries in horses undergoing general anesthesia. Factors identified in other studies, such as hypoxemia, hypotension, or the ASA score, were not related to recovery quality in our study. These findings collectively emphasize the multifactorial nature of anesthetic recovery in horses, emphasizing that recovery quality remains difficult to predict. Our results advocate for further investigation into anesthetic practices to enhance recovery quality, aiming to reduce mortality rates and improve overall equine-patient outcomes.

## Figures and Tables

**Table 1:** Recovery score according to the CEPEF and recovery score according to our cut-off value for the study.

Recovery Score Recorded in the CEPEF Form	Recovery Scoring Assigned in This Study
1. One attempt to stand, no ataxia. 2. One-to-two attempts to stand, some ataxia.	Good recovery
3. >2 attempts to stand but quiet recovery 4. >2 attempts to stand, excitation 5. Severe excitation. Equine-patient injured	Bad recovery

**Table 2:** Demographic, procedural, and anesthesia-related complications in 1057 horses undergoing general anesthesia with recovery outcomes classified as “good” or “bad” based on recovery scores.

Variable	Good Recovery (n = 873)	Bad Recovery (n = 184)	Total (n = 1057)
Mean body weight (kg)	496.4	506.3	498.3
Age (months)	123.3	155.7	128.9
Sex:			
Stallion	185	26	211
Gelding	363	75	439
Non-pregnant female	315	77	392
Pregnant female	10	5	15
Breed:			
Warmbloods	416	81	497
Racehorse breeds	75	29	105
Draft horse breed	76	13	89
Pony	73	6	79
American horse breeds	33	16	49
Baroque horse breeds	81	12	93
Other breeds	132	23	145
Emergency:			
Yes	206	83	289
No	667	100	768
ASA score:			
I	150	12	162
II	535	92	628
III	130	39	169
IV	50	35	85
V	8	5	13
Procedure:			
Diagnostic Imaging	8	1	9
Colic surgery	152	75	227
Abdominal surgery	26	2	28
Ophthalmology	159	26	185
Fracture repair	10	2	12
Orthopedic surgery	173	31	205
Urogenital	152	19	171
Ear/Nose/Throat	23	4	27
Miscellaneous	229	33	262
Complications:			
Hypotension	499	113	613
Hypoxemia	111	25	136
Hypercapnia	27	10	37
Drugs:			
Dobutamine	534	129	663
Acepromazine	653	42	695
Morphine	156	22	178
Salbutamol	137	52	189
Times (mean)	Good recovery (n = 873)	Bad recovery (n = 184)	Total (n = 1057)
Duration anesthesia (minutes):	112	149.9	118.6
Duration surgery (minutes):	73.9	105.8	79.4
Recovery Time (minutes):	44.3	77.6	50.0

**Table 3:** Breed pairwise analysis by chi squared testing. A positive sign indicates the breeds in the left column have worse recoveries than those in the top row, and a negative sign indicates breeds in the left column have better recoveries than those in the top row. WB = Warmblood, Race = Racehorse, Draft = Draft-bred horse, American = American horse breed.

	Baroque	Draft	Mixed	Pony	Race	American	WB
Baroque	X	*p* = 0.906	*p* = 0.382	*p* = 0.377	−*p* = 0.00176	−*p* = 0.00960	*p* = 0503
Draft	*p* = 0.906	X	*p* = 0.614	*p* = 0.235	−*p* = 0.0436	−*p* = 0.0231	*p* = 0.808
Mixed	*p* = 0.382	*p* = 0.614	X	*p* = 0.0534	*p* = 0.101	*p* = 0.0525	*p* = 0.7105
Pony	*p* = 0.377	*p* = 0.235	*p* = 0.0534	X	−*p* = 0.0121	−*p* = 0.000646	*p* = 0.0662
Race	+*p* = 0.0176	+*p* = 0.0436	*p* = 0.101	+*p* = 0.0121	X	*p* = 0.653	+*p* = 0.00964
American	+*p* = 0.00960	+*p* = 0.0231	*p* = 0.0525	+*p* = 0.000646	*p* = 0.653	X	+*p* = 0.0777
WB	*p* = 0.503	*p* = 0.808	*p* = 0.7105	*p* = 0.0662	−*p* = 0.00964	−*p* = 0.00777	X

## Data Availability

Raw data are available via Sandersen, Charlotte (2025). Rawdata Bienve study. Figshare. Dataset at https://doi.org/10.6084/m9.figshare.28323779.v1.
